# A Machine Learning Predictive Model for Post-Ureteroscopy Urosepsis Needing Intensive Care Unit Admission: A Case–Control YAU Endourology Study from Nine European Centres

**DOI:** 10.3390/jcm10173888

**Published:** 2021-08-29

**Authors:** Amelia Pietropaolo, Robert M. Geraghty, Rajan Veeratterapillay, Alistair Rogers, Panagiotis Kallidonis, Luca Villa, Luca Boeri, Emanuele Montanari, Gokhan Atis, Esteban Emiliani, Tarik Emre Sener, Feras Al Jaafari, John Fitzpatrick, Matthew Shaw, Chris Harding, Bhaskar K. Somani

**Affiliations:** 1 Department of Urology, University Hospital Southampton, Southampton SO16 6YD, UK; ameliapietr@gmail.com; 2 Department of Urology, Freeman Hospital, Freeman Road, Newcastle-upon-Tyne NE1 7DN, UK; robgeraghty@btinternet.com (R.M.G.); r.veeratterapillay@nhs.net (R.V.); Alistair.rogers2@nhs.net (A.R.); john.fitzpatrick4@nhs.net (J.F.); Matthew.shaw7@nhs.net (M.S.); c.harding@nhs.net (C.H.); 3 Department of Urology, University of Patras, 26504 Patras, Greece; pkallidonis@yahoo.com; 4 IRCCS Ospedale San Raffaele, Urology, 20019 Milan, Italy; lucavilla01984@gmail.com; 5 Department of Urology, IRCCS Fondazione Ca’ Granda-Ospedale Maggiore Policlinico, University of Milan, 20019 Milan, Italy; dr.lucaboeri@gmail.com (L.B.); montanari.emanuele@gmail.com (E.M.); 6 Department of Urology, Faculty of Medicine, Istanbul Medeniyet University, Istanbul 34720, Turkey; gokhanatis@hotmail.com; 7 Department of Urology, Fundació Puigvert, 08001 Barcelona, Spain; emiliani@gmail.com; 8 Department of Urology, Marmara University, Istanbul 34720, Turkey; dr.emresener@gmail.com; 9 Victoria Hospital, Kirkcaldy KY1 2ND, UK; feras.al.jaafari@gmail.com

**Keywords:** kidney stones, urosepsis, ureteroscopy, laser lithotripsy, urolithiasis, nephrolithiasis, kidney calculi, predictor factors

## Abstract

Introduction: With the rise in the use of ureteroscopy and laser stone lithotripsy (URSL), a proportionate increase in the risk of post-procedural urosepsis has also been observed. The aims of our paper were to analyse the predictors for severe urosepsis using a machine learning model (ML) in patients that needed intensive care unit (ICU) admission and to make comparisons with a matched cohort. Methods: A retrospective study was conducted across nine high-volume endourology European centres for all patients who underwent URSL and subsequently needed ICU admission for urosepsis (Group A). This was matched by patients with URSL without urosepsis (Group B). Statistical analysis was performed with ‘R statistical software’ using the ‘randomforests’ package. The data were segregated at random into a 70% training set and a 30% test set using the ‘sample’ command. A random forests ML model was then built with *n* = 300 trees, with the test set used for internal validation. Diagnostic accuracy statistics were generated using the ‘caret’ package. Results: A total of 114 patients were included (57 in each group) with a mean age of 60 ± 16 years and a male:female ratio of 1:1.19. The ML model correctly predicted risk of sepsis in 14/17 (82%) cases (Group A) and predicted those without urosepsis for 12/15 (80%) controls (Group B), whilst overall it also discriminated between the two groups predicting both those with and without sepsis. Our model accuracy was 81.3% (95%, CI: 63.7–92.8%), sensitivity = 0.80, specificity = 0.82 and area under the curve = 0.89. Predictive values most commonly accounting for nodal points in the trees were a large proximal stone location, long stent time, large stone size and long operative time. Conclusion: Urosepsis after endourological procedures remains one of the main reasons for ICU admission. Risk factors for urosepsis are reasonably accurately predicted by our innovative ML model. Focusing on these risk factors can allow one to create predictive strategies to minimise post-operative morbidity.

## 1. Introduction

Kidney stones disease (KSD) has seen an increase in incidence and prevalence over the last few decades [1,2,3,4]. This can vary according to the ethnicity, geographical origin and weather along with diet and behavioural variations such as exercise, diet and fluid intake [2,3]. Treatment options consist of shockwave lithotripsy (SWL), ureteroscopy and laser stone lithotripsy (URSL) and percutaneous nephrolithotomy (PCNL) in accordance with the stone size and location [2,3].

URSL is becoming an increasingly common procedure to treat kidney and ureteral stones. There has been an upwards trend of URSL over the last few years, becoming a popular surgical procedure for KSD [4]. Despite being minimally invasive in nature, the use of high-pressure irrigation and the dispersion of potential infected stone particles can cause urinary tract infections (UTIs), and, in rare cases, it can cause severe systemic infection and sepsis. Post-ureteroscopic infectious complications and urosepsis are uncommon but serious life-threatening complications and range from 2.2% to 20% in several studies [5]. They affect the immunological system but also coagulation, the central nervous system, the autonomic nervous system, the endocrine system, the cardiovascular system, the liver and the kidneys [6].

The term systemic inflammatory response syndrome (SIRS) has been previously used along with the term severe sepsis and septic shock. While the SIRS criteria include fever, tachycardia, tachypnoea and raised serum inflammatory markers, having two or more of these is called sepsis. The sequential organ failure assessment (SOFA) score, which is an index of organ dysfunction secondary to infection, was used to predict ICU mortality based on laboratory results and clinical data. High SOFA score immediately correlates to the risk of mortality [7]. The predictive value of the SOFA score for in-hospital mortality was superior to that of the SIRS criteria. The Third International Consensus Definitions for ‘Sepsis and Septic Shock’ (sepsis 3) updated the definition of sepsis [8]. The presence of >2 criteria were identified under quick SOFA (qSOFA) score.

Sepsis can also present as septic shock characterised by severe cardio-circulatory compromise, requiring multiorgan support, adequate fluid resuscitation and intensive care unit (ICU) support [9]. Management of sepsis includes intervention at multiple levels, from administration of antibiotics to fluid resuscitation, hemofiltration, cardiovascular and respiratory support. Furthermore, the long-term social, physical, psychological and cognitive disabilities of patients who survive sepsis require huge healthcare and social support with consequent economic impact [10].

Severe urosepsis can lead to multiorgan failure and death. Mortality secondary to ureteroscopy has risen over the past decade. In a recent systematic review, the cause of death after URSL for stone disease was found to be sepsis in over half of all reported patients [11]. The aim of our paper was to analyse the predictors for severe urosepsis in patients that needed ICU admission. We used a matched ureteroscopy cohort, with which we built a machine learning (ML) model to predict which patients would develop urosepsis needing ICU treatment.

## 2. Methods

A retrospective study was conducted across 9 high-volume endourology European centres from 5 countries (Italy, Greece, Turkey, Spain and the UK). The inclusion criteria were all patients who underwent URSL for stone disease and subsequently developed urosepsis that needed ICU admission (Group A). This was matched by a similar group of patients who had a URSL procedure for stone disease without urosepsis (Group B). The data on patient demographics, comorbidities, ASA grade, previous history of UTIs, prior endoscopic procedures, pre-operative urine culture and laboratory parameters for infection both pre- and post-surgery were collected over an 11-year period from these centres between 2009 and 2020.

While the study included patients who developed urosepsis that needed ICU admission, patients with non-infectious complications and not needing ICU were excluded. Urinary tract infection was defined as a positive urine culture with >104 colony forming units per millilitre (CFU)/mL. Information on empirical and selective antibiotics used was also collected. Further variables were analysed with particular attention towards stent dwell time, intraoperative use of ureteral access sheath (UAS) and operative time. Primary and secondary outcomes were complication and stone-free rates (SFR), respectively. Cases were matched with the control group (Group B) for age; gender; and comorbidities known to increase the risk of post-ureteroscopic UTI: diabetes mellitus (DM), immunosuppression, neurological disorders, previous urinary tract reconstruction and abnormal upper tract anatomy [12].

Statistical analysis was performed using R (R statistical software, Vienna, Austria) using the ‘randomforests’ package. The data were segregated at random into a 70% training set and a 30% test set using the ‘sample’ command with the seed set at 1234. A random forests machine learning model was then built with *n* = 300 trees, with the test set used for internal validation. A random forests model generates a set number (i.e., 300 in this case) of random decision trees, which are then aggregated to form the single model. Diagnostic accuracy statistics (sensitivity, specificity and area under the curve) for model performance were generated using the ‘caret’ package. Graphs were generated using ‘ggplot2′, and these include a receiver operator curve (ROC) for the model, along with a ‘mean decrease gini’ plot (demonstrates variables ranked according to how frequently they are represented in the random trees prior to aggregation—more important variables will be represented more frequently). Explanatory graphs with individual predictions are presented following generation with the ‘lime’ (local interpretable model agnostic explanations) package. The model was deployed as a ‘shiny’ application using the ‘shiny’ package.

## 3. Results

A total of 114 patients were included (57 in each group) with a mean age of 60 years (±16) with a male:female ratio of 1:1.19 in both groups (Table 1).

The numbers of patients in Groups A and B with DM (*n* = 15, 26.3% and *n* = 12, 21.1%), immunocompromise (*n* = 3 and 1), neurological disorder (*n* = 1 and 1), previous urinary tract reconstruction (*n* = 1 and 0) and abnormal upper tract anatomy (*n* = 1 and 5) were as shown. There were 14 (24.6%) and 3 (5.3%) patients with a history of UTI for Groups A and B, respectively. Indwelling stent dwell time for Groups A and B were 52 ± 63 days and 30 ± 60 days for 33 and 26 patients, respectively. In each group, 31 patients (54.3%) had a single stone; the remaining (45.6%) had more than one stone (range: 2–5). The single largest stone sizes in Groups A and B were 10 ± 5 mm and 8 ± 4 mm, respectively. In both groups, 15 patients (26.3%) had a pre-operative positive urine culture that was treated as per local protocol. The mean operative time was 58 ± 31 min and 43 ± 23 min, and the SFR was 48.6% and 89.5% in Groups A and B, respectively. One patient in Group A (83-year-old female) died from urosepsis. She also had a history of prior recurrent UTIs, was ASA 3 and suffered with Alzheimer’s dementia. She was not pre-stented, no access sheath was used, and a procedural time of 45 min with a post-operative stent left in situ was noted. She developed multi-resistant *Escherichia coli* infection and died of septic shock after 2 days.

**Figure 1:**
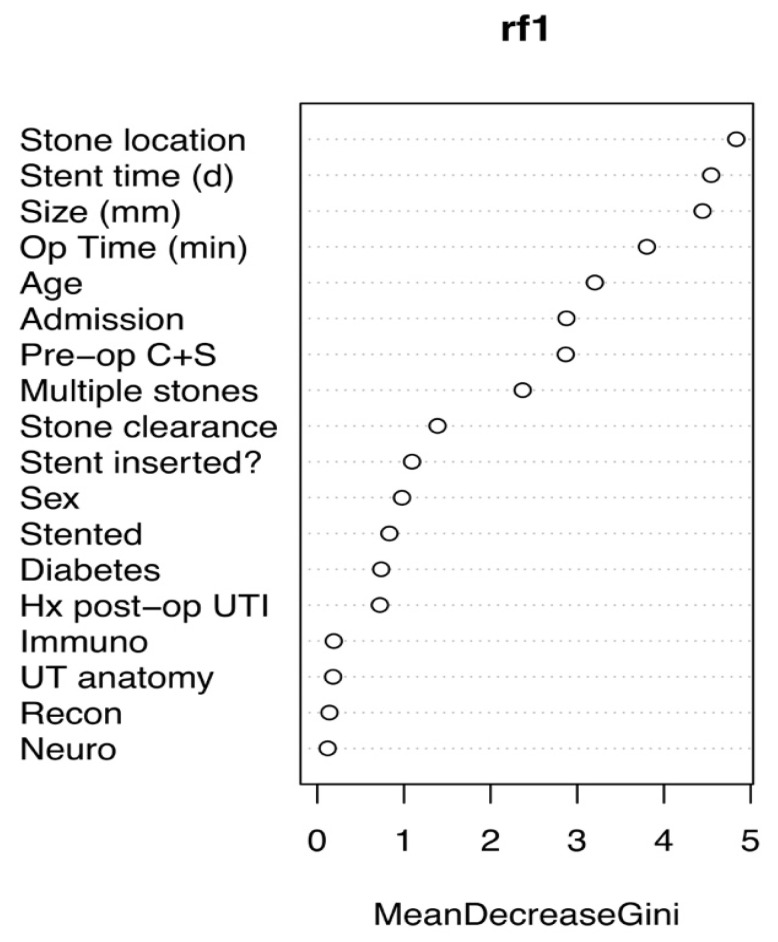
Gini is a graph of factors most commonly represented in the random trees (*n* = 300 trees) produced prior to tree aggregation to form the model. The more frequently the variable is represented, the more important the variable will be to the final model.

**Figure 2:**
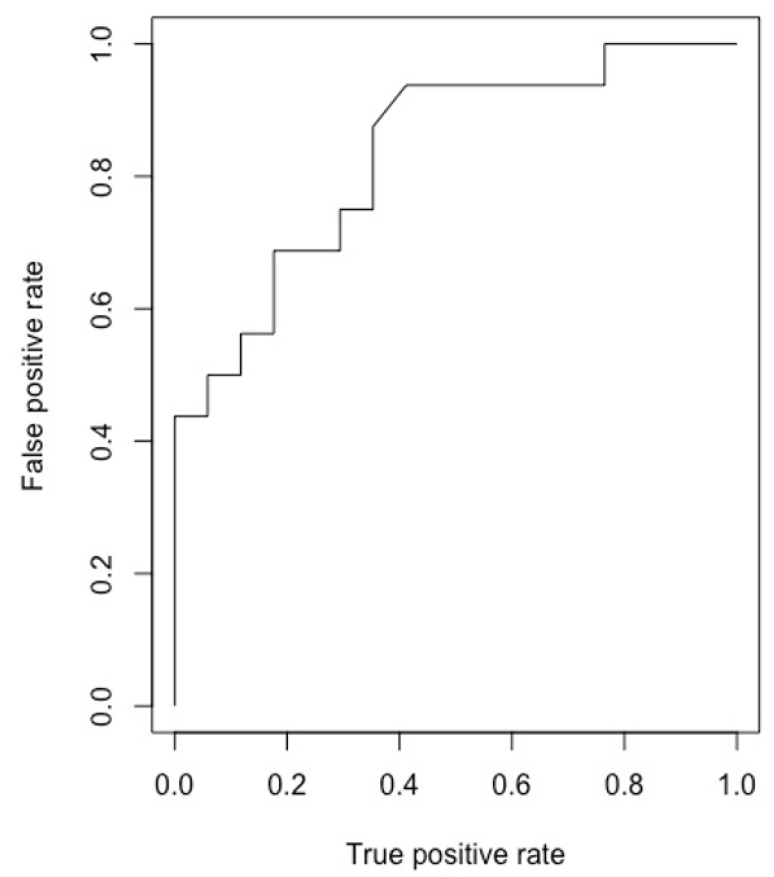
Receiver operator curve (ROC) for internally validated model.

**Figure 3:**
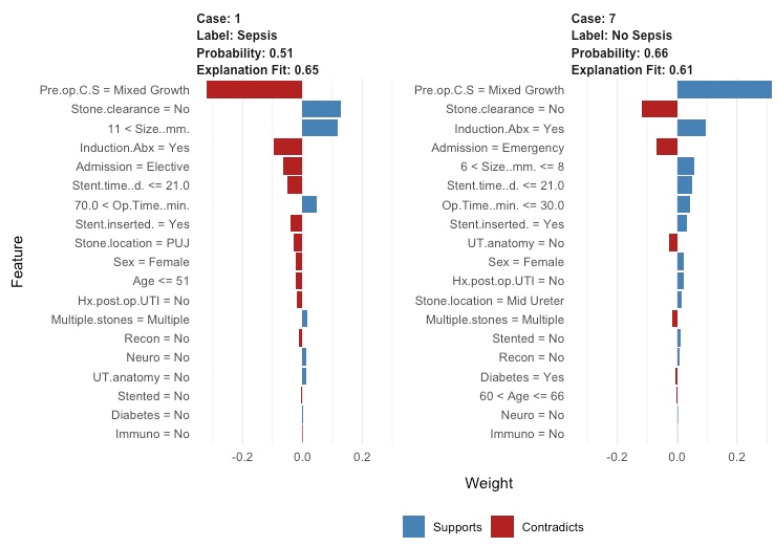
Lime (local interpretable model-agnostic explanations graphs) deployed for the model. Predictions are given on a case-by-case basis, along with the explanatory variables contributing to that outcome, within the context of the model.

The ML model correctly predicted risk of sepsis in 14/17 (82%) cases (Group A) and predicted those without urosepsis for 12/15 (80%) controls (Group B), whilst, overall, it also discriminated between the two groups, predicting both those with and without sepsis. Our model accuracy was 81.3% (95%, CI: 63.7–92.8%), sensitivity = 0.80, specificity = 0.82 and area under the curve = 0.89. Predictive values most commonly accounting for nodal points in the trees were large proximal stone location, long stent time, large stone size and long operative time (Figure 1 and Figure 2). The model was deployed onto the internet using the ‘shiny’ application. Users are able to input patient, stone and operative characteristics for an outcome prediction. The outcome prediction is either ‘sepsis’ or ‘no sepsis’ and is presented using the ‘lime’ package, which demonstrates which variables are affecting the outcome most within the context of the model (see Figure 3 and https://endourology.shinyapps.io/Urosepsis_Predictor/, accessed on 22 August 2021).

## 4. Discussion

### 4.1. Meaning of the Study

In the current study, we used data collected from different centres across Europe to develop an easy-to-use machine learning tool for prediction of post-operative sepsis and ICU admission in patients undergoing elective URSL for stone disease. Using a machine-learning approach, we found that proximal stone location, long stent dwelling time, large stone size and long operative time can reasonably accurately identify patients at risk of developing post-operative urosepsis.

All predictive parameters analysed in our model are part of the routine assessment to identify the indication for surgery, and this makes our model accessible to all urologists. Preoperative identification of those patients who have a higher risk of developing sepsis or requiring post-operative ICU can help to create preventative strategies such as focusing on antibiotic prophylaxis, preoperative counselling and intraoperative support. This may also prevent exposure of low-risk patients to unnecessary antibiotic therapy.

### 4.2. Risk Factors of Post-Ureteroscopic Urosepsis from Previous Published Literature

This topic has been the subject of heated debate in the last few years, with many published studies attempting to identify common risk factors of post-operative urosepsis. However, no other studies to date have used a machine learning model to predict risk factors of urosepsis. A recent study by Bhanot et al. identified urosepsis with a higher risk of death after URSL procedures [11]. Predictors identified in their systematic review allowed the creation of recommendations, such as preoperative urine culture and appropriate treatment; reducing the operative time; trying to favour staged procedures, especially in patients with large stone burden; and minimising stent dwell time. Care in preoperative assessment and postoperative monitoring was identified as a strategy for early detection of complications and minimising the risk of mortality.

A recent study demonstrated individual risk factors for urosepsis [12]. Chugh et al. carried out a systematic review of the literature to identify predictors of infectious complications following URSL for stone disease. Patients with multiple comorbidities, such as obesity, old age, female gender, neurogenic bladder, long operative time and indwelling ureteric stents, were shown to be related to a higher risk for UTIs or sepsis. Strategies such as prophylactic antibiotics, limiting stent dwell or procedural time and staging procedures were identified as possible preventative measures. Similar parameters were identified by Southern et al. [13], who retrospectively analysed 3298 patients undergoing URSL for stone disease and found that 7% of them developed post-operative SIRS/febrile UTIs. In their multivariate logistic regression, the authors found that female gender, surgical time and positive preoperative urine culture were predictors for infectious complications.

Prior emergency decompression for infected obstructed kidney may appear as a possible risk factor for urosepsis. However, in a study by Pietropaolo et al., only 1.2% developed sepsis after elective stone removal in such patients [14], demonstrating that initial septic presentation is not a risk factor for post-operative urosepsis when it comes to elective URSL. Martov et al., on behalf of CROES group [15], collected data from 1325 patients who underwent URSL for renal and ureteric stones. They identified predictive factors of postoperative UTI and fever as female gender, Crohn’s disease, cardiovascular disease, high stone burden and an ASA score of 2 or higher.

A Chinese group in a study conducted by Xu et al. [16] studied the trend of the serum parameter bone morphogenetic protein endothelial cell precursor-derived regulator (BMPER) in patients with urosepsis following ureteroscopic stone treatment. They concluded that a high BMPER concentration is a strong predictor of adverse outcome in patients with post-operative urosepsis. In their meta-analysis, Bhojani et al. [17] found six risk factors statistically associated with increased postoperative urosepsis risk, such as preoperative stent, positive preoperative urine culture, ischaemic heart disease, older age, longer procedure time and diabetes mellitus. Bai et al. retrospectively reviewed 1421 patients who underwent ureteroscopy and stone laser treatment and found that patients with positive preoperative urine culture or long operation duration had a higher risk of developing urosepsis after URSL [18].

### 4.3. Comparison with Other ML Studies

The role of artificial intelligence (AI) in medicine is expanding day-by-day due to its capability of performing human cognitive tasks. The huge amount of data extracted by the electronic medical records can be used for computer-based predictions that can help in improving patient care [19]. There are four subfields of AI in health care, and machine learning (ML) is one of them. This is a technique that uses algorithms and allows a computer to recognise patterns and learn automatically through experience and by the use of data. The method is being increasingly used in all medical specialties, including urology, and its use is already widespread in all urological subspecialties. Song et al. [20] in their review assessed whether ML models were superior compared to logistic regression (LR), a more conventional prediction model. They used both techniques in predicting acute kidney injury (AKI) and agreed that in the literature, ML was superior due to its more variable and adaptable performance.

Aminsharifi et al. [21] analysed data of 146 adult patients who underwent percutaneous nephrolithotomy (PCNL) to validate the efficiency of an ML algorithm for predicting the outcomes after PCNL. This program predicted the PCNL results with an accuracy of up to 95%. Blum et al. [22] created an ML framework to improve the early detection of clinically significant hydronephrosis caused by pelvic–ureteric junction obstruction based on data from renograms. This had a 93% accuracy in predicting earlier detection of severe cases requiring surgery.

ML is also utilised in cancer diagnosis or treatment outcomes. Kocak et al. [23] developed models for distinguishing three major subtypes of renal cell carcinomas (RCC) using an ML model based on CT scan results. The model could satisfactorily distinguish non-RCC from RCC. Similarly, Feng et al. [24] used a ML approach to accurately discriminate between small angiomyolipoma (AML) and RCC in CT scans with high accuracy, sensitivity and specificity. Hasnain et al. [25] used an ML algorithm to predict cancer recurrence and survival after radical cystectomy based on imaging, operative findings and pathology. Deng et al. [26] developed an ML algorithm that could differentiate metastatic castrate-resistant prostate cancer patients in two groups, those who could tolerate docetaxel and those who could not. This model managed to predict therapeutic failure in patients who could potentially develop toxic effects of docetaxel chemotherapy.

### 4.4. Strengths, Limitations and Areas of Future Research

The machine learning models provide a new benchmark for predicting surgical or oncological outcomes and highlight opportunities for improving care using optimal preoperative and operative data collection. The limitation of our study is based on its retrospective nature. Further prospective and randomised controlled trials are required to corroborate our findings and to be able to write specific recommendations that will allow the prediction of post-URSL sepsis. Furthermore, external validation of our ML model is required to confirm its effectiveness and predictive power, with subsequent development of a mobile-phone app to be used in day-to-day clinical practice.

Genetics has recently been introduced as a new field of research on the topic by Giamarellos-Bourboulis et al. [27]. They have related low concentrations of immunoglobulins with adverse outcomes in urosepsis response. Carriage of minor genetic deficiency in antibody production can be related to poor sepsis prognosis. This field has not been fully exploited to date, but a genomic approach should be taken into consideration in the future as an aid to identify the origin of this deadly disease.

Urosepsis requiring ICU support is a rare post-operative event and, despite multiple centres involved in data collection, only few cases were available for analysis. AI and ML models are certainly expected to play an increasing role in the medical field due to the global technological advancement and their capability of learning and reproducing tasks without instructions. However, the topic is complex, and issues exist regarding the reliability of machine diagnosis, the consent for data sharing and the external control of large industries or data holders with the inherent conflict of interest this brings. Nevertheless, future applications of ML models are yet to come, and the use of these algorithms can only increase.

## 5. Conclusions

Urosepsis after endourological procedures, such as URSL, remains one of the main causes for ICU admission and consequent post-operative disabilities or mortality. Risk factors for urosepsis are reasonably accurately predicted by our innovative machine learning model. Focusing on these risk factors can allow one to create predictive strategies to minimise post-operative morbidity. External validation of the model is required to confirm its effectiveness in predicting sepsis.

## Figures and Tables

**Table 1:** Patient characteristics of both Groups A and B.

		Group A, *n* = 57	Group B, *n* = 57
Mean age (years) ± SD		60 ± 16	60 ± 16
Male gender, *n* (%)		26 (45.6%)	26 (45.6%)
Diabetes, *n* (%)		15 (26.3%)	12 (21.1%)
Immunosuppression/modulation, *n* (%)		3 (5.3%)	1 (1.8%)
Neurological disorder, *n* (%)		1 (1.8%)	1 (1.8%)
Previous urinary tract reconstruction, *n* (%)		1 (1.8%)	0
Abnormal upper tract anatomy, *n* (%)		1 (1.8%)	5 (8.8%)
History of recurrent UTI, *n* (%)		14 (24.6%)	3 (5.3%)
Emergency admission		30 (52.6%)	9 (15.8%)
Presence of pre-operative stent, *n* (%)		33 (57.9%)	26 (45.6%)
Mean stent dwell time (days) ± SD		52 ± 63	30 ± 60
Number of stones	1	31	31
	2	20	13
	3	3	13
	4	2	0
	5	1	0
Mean largest stone diameter (mm) ± SD		10 ± 5	8 ± 4
Location, *n*	Vesicoureteric junction (VUJ)	3	3
	Distal ureter	7	11
	Mid ureter	8	11
	Proximal ureter	8	13
	Renal	31	15
	N/A	0	4
Positive pre-operative urine culture, *n* (%)		15 (26.3%)	15 (26.3%)
Mean operative time (mins) ± SD		58 ± 31	43 ± 23
Post-operative stent insertion, *n* (%)		36 (46.2%)	42 (53.8%)
Stone free, *n* (%)		34 (48.6%)	51 (89.5%)

## Data Availability

As data is identifiable, it will not be made available as per ethical approval.
